# Effect of particle size distribution on the electrochemical performance of micro-sized silicon-based negative materials†

**DOI:** 10.1039/c8ra00539g

**Published:** 2018-02-23

**Authors:** Shuaijin Wu, Bing Yu, Zhaohui Wu, Sheng Fang, Bimeng Shi, Juanyu Yang

**Affiliations:** General Research Institute for Nonferrous Metals Beijing 100088 China juanyuyang@163.com; China Automotive Battery Research Institute Co., Ltd Beijing 100088 China

## Abstract

Si has been extensively examined as a potential alternative to carbonaceous negative materials, because it shows exceptional gravimetric capacity and abundance. In recent years, the strategy of using nano-structured silicon materials as building blocks to build micro-sized silicon-based materials has been widely studied. In this work, a commercialized and benchmark micro-sized silicon-based material (denoted as SiO_*x*_/C) is used as research target and three groups of materials with different particle size distributions (PSDs) were obtained by simply mechanical sieving. The effects of PSD on the electrochemical performance and electrode structure of micro-sized silicon-based negative electrodes are discussed. The optimized selection of micro-sized active material PSD presents a comprehensive way for developing and characterizing Si-based negative electrodes for practicable high-energy LIBs. In this case, the optimized SiO_*x*_/C composite electrode with a particle size of 22.7 μm and narrow PSD shows enhanced cycling stability with a high capacity retention of 84.31% over 100 cycles.

## Abstract 2

Si has been extensively examined as a potential alternative to carbonaceous negative materials, because it shows exceptional gravimetric capacity and abundance.

## Graphical abstract

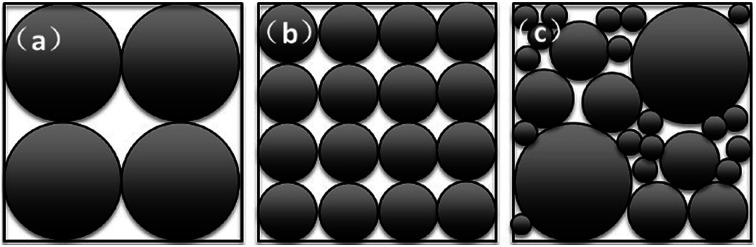

## 1. Introduction

**Fig. 1:**
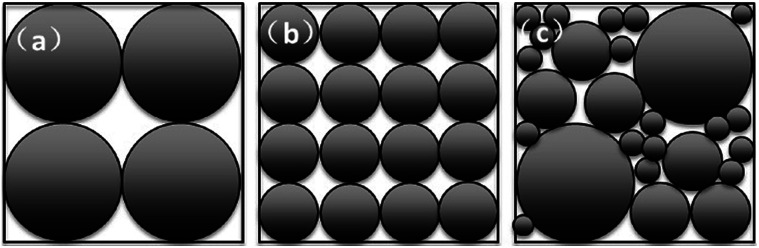
Schematic of pore structure with different particle size.

Silicon has a much higher electrochemical capacity (3579 mA h g^−1^ for Li_15_Si_4_ alloy formed at room temperature) than commercial graphite materials (372 mA h g^−1^) and shows great prospects for development.^1,2^ Nano-silicon materials effectively avoid the pulverization of the material during cycling with shorter Li^+^ and electron transport paths. However, large surface area and low tap density limit the application of nano-silicon materials in practical production.^3–7^ Numerous approaches for the advance of Si negative electrodes have been reported, however, in order to achieve the practical application of negative electrode materials, further detailed analyses and feasibility studies are required. The strategy of using nano-structured silicon materials as building blocks to construct micro-sized silicon-based materials has been proposed and discussed in recent years.^7–12^ However, the effect of micro-sized silicon-based active material particle size distribution on the electrochemical performance is rarely studied. In the silicon/carbon electrode system, active materials are main body of electrode structure which could reach the range of 80–95%. The particle size distribution and particle morphology directly affect the final pore structure which is closely related with the electrode reaction and the volume change process. Therefore, investigating the relationship between the basic properties of the active material and the electrode pore structure is necessary and practical significant, cause that it contributes to optimizing the electrode active materials. In theory, when assuming that the particles are accumulated in tangential state and all particles are uniform in size, as shown in Fig. 1(a) and (b), the particle diameter is *D* and *D*/2, respectively. The effect of the active particles accumulation with equally particle size on the pore structure is mainly manifested in the change of pore size while the change of porosity is not significant (remain (8*D*^3^ − 4/3π*D*^3^)/8*D*^3^). However, when the particle size of the active material distributes in a certain range (as shown in Fig. 1(c)), the further stacking between the particles narrows the pore size, reduces the porosity and finally improves the space utilization. It should be pointed out that the size of the pores is inversely proportional to tortuosity, that is, the smaller the pore size, the higher the tortuosity, causing more difficulty in corresponding ions diffusion in liquid phase. In consequence, analysis on difference of electrochemical performance from solid state porous electrode perspective is crucial to optimize material PSD range suitable for practical LIBs.

Herein, we used a commercialized and benchmarking micro-sized silicon-based material (denoted as SiO_*x*_/C) as research target, the battery cycling performance and electrode morphological investigation based on different PSDs of materials will be mainly discussed. A reasonable guide and a meaning insight in the application of micro-sized Si negative materials will be presented for other researchers.

## 2. Experimental

Three groups of SiO_*x*_/C materials with different PSD was obtained by mechanical sieving and compared *via* electrochemical properties as well as electrode structure. Material characterizations are conducted by diverse analytical tools. The morphologies of the materials and electrodes were investigated by SEM (Hitachi S4800, Japan). PSD was measured using a laser particle size analyzer (Malvern, Mastersizer 3000). The mass ratio of the active materials, conductive agent and binder was 86 : 6 : 8. Water-soluble styrene butadiene rubber–carboxymethyl cellulose–polyacrylic acid (SBR–CMC–PAA) binder composition (total 8.0 wt%, CMC : PAA : SBR = 4 : 0.5 : 5.5) and Super-P/VGCF (5 : 1) were used as binder and conductive agent respectively. The obtained slurry was pasted onto the copper foil with the active material layer thickness of 200 μm. After drying at 25 °C for 3 h, the film was cut into a circular shape (14 mm in diameter) and calendared at electrode density of 1.1 g cm^−3^. In this study, the areal density of electrode was around 4 mg cm^−2^. After drying the electrodes in a vacuum oven at 100 °C for 9 h, CR2032-type half coin cells were assembled in an Ar-filled glove box (O_2_ < 0.1 ppm, H_2_O < 0.1 ppm). The above electrode and Li foil acted as the working electrode and counter electrode. Celgard 2300 membrane was used as separator and 1 M LiPF_6_/EC : DEC : EMC (volume ratio = 1 : 1 : 1) was used as electrolyte. To characterize the battery performance of SiO_*x*_/C with different PSDs, electrochemical evaluation was performed in the segmented constant current discharge mode (0.1C for 0.01–1.5 V, 0.01C for 0.005–0.01 V). The porosity of the SiO_*x*_/C composite electrodes before cycling were further analysed by mercury intrusion testing. Several critical factors that could affect the electrode performance were investigated, such as cycling behaviour, cycling CE and changes in the electrode structure before and after cycling. The electrochemical impedance spectroscopy (EIS) measurements were performed on a Metrohm PGSTAT 302N electrochemical workstation with frequencies ranging from 10 kHz to 10 mHz and an AC signal of 5 mV in amplitude as perturbation. All the tests were performed at room temperature.

## 3. Results and discussion

### 3.1. Characterization of the SiO_*x*_/C samples with different PSDs

The SiO_*x*_/C material used was purchased from BTR New Energy Co., Ltd., which is one of common commercially available silicon-based materials. The original powder material SiO_*x*_/C denoted as BSC0 was divided into three groups by shaking through 325 mesh, 400 mesh and 500 mesh standard screen, numbering BSC2, BSC3, BSC4 according to the order from large to small. The PSD of active materials could significantly affect the structure of the electrode. The average particle size (*D*_50_) and the PSD shape (width and peak shape) are main factors representing the particle size distribution. Flat and wide PSD curves represent uneven size distribution range while tall and narrow curves show more uniform particle size distribution. The *D*_50_ of BSC0 is 20.1 μm, while those of BSC2, BSC3, BSC4 are confirmed as 22.7 μm, 15.7 μm, 13.5 μm respectively, size decreasing in turn. BSC0 and BSC2 have comparable *D*_50_ while BSC0 has a slightly wider size distribution than BSC2 (with *D*_90_/*D*_50_ values of 1.7 and 1.6 respectively), indicating that size distribution of BSC2 is more homogeneous relative to BSC0. Proper particle size distribution and high tap density could promote the compatibility between the active material and conductive agent, contributing to a more uniform slurry and increasing the volumetric energy density of electrodes.^13^

**Fig. 2:**
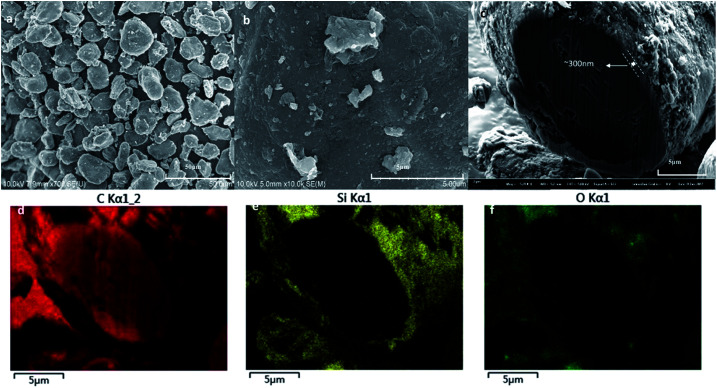
SEM images of the original SiO_*x*_/C material (BSC0) at different magnifications (a, b), the cross-sectional image of SiO_*x*_/C composite (c), together with corresponding EDS elemental mapping results for C (d), Si (e), and O (f).

**Fig. 3:**
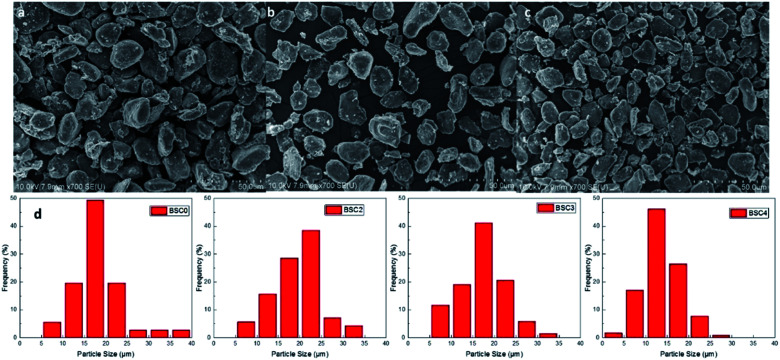
SEM images of the SiO_*x*_/C with different PSDs (a–c) and corresponding particle size statistics results (d).

Fig. 2 shows the scanning electron microscopy (SEM) images of original SiO_*x*_/C material BSC0 at different magnifications. As presented in Fig. 2(a and b), BSC0 shows regularly spherical shaped with relatively smooth surface texture. The BSC0 material particle size distribution is wide with large particle size up to 40 μm and small particles only a few microns. As shown in the cross-sectional FIB-SEM image (Fig. 2(c)), the SiO_*x*_/C particle presents a clear core–shell structure. The corresponding X-ray energy dispersive spectroscopy (EDS) detects the distribution of Si, C and O elements (as shown in Fig. 2(d–f)). Through EDS mapping analysis, it is confirmed that the coating layer on the micro-sized carbon particle surface is silicon layer (∼300 nm). It is presumed that the small particles ranging from 1 μm to 5 μm attached to the surface (as shown in Fig. 2(b)) are SiO_*x*_ aggregates. SEM images and corresponding Nano Measurer analysis results of the SiO_*x*_/C materials with different PSDs are shown in Fig. 3. According to the low magnification SEM image of BSC2 (Fig. 3(a)), compared with BSC0, BSC2 material particle size is more uniform and mainly composed of spherical particles between 20 μm and 25 μm. Particle size of BSC3 is further reduced to ∼15 μm (Fig. 3(b)) while the sphericity is not as well as that of front samples. BSC4 material (Fig. 3(c)) is mainly composed of 10–15 μm particles, showing the worst sphericity and the majority particles showing ellipsoidal shape. The statistics particle size histogram of the SiO_*x*_/C materials is shown in Fig. 3(d).

### 3.2. Electrochemical testing of SiO_*x*_/C samples with different PSD

**Fig. 4:**
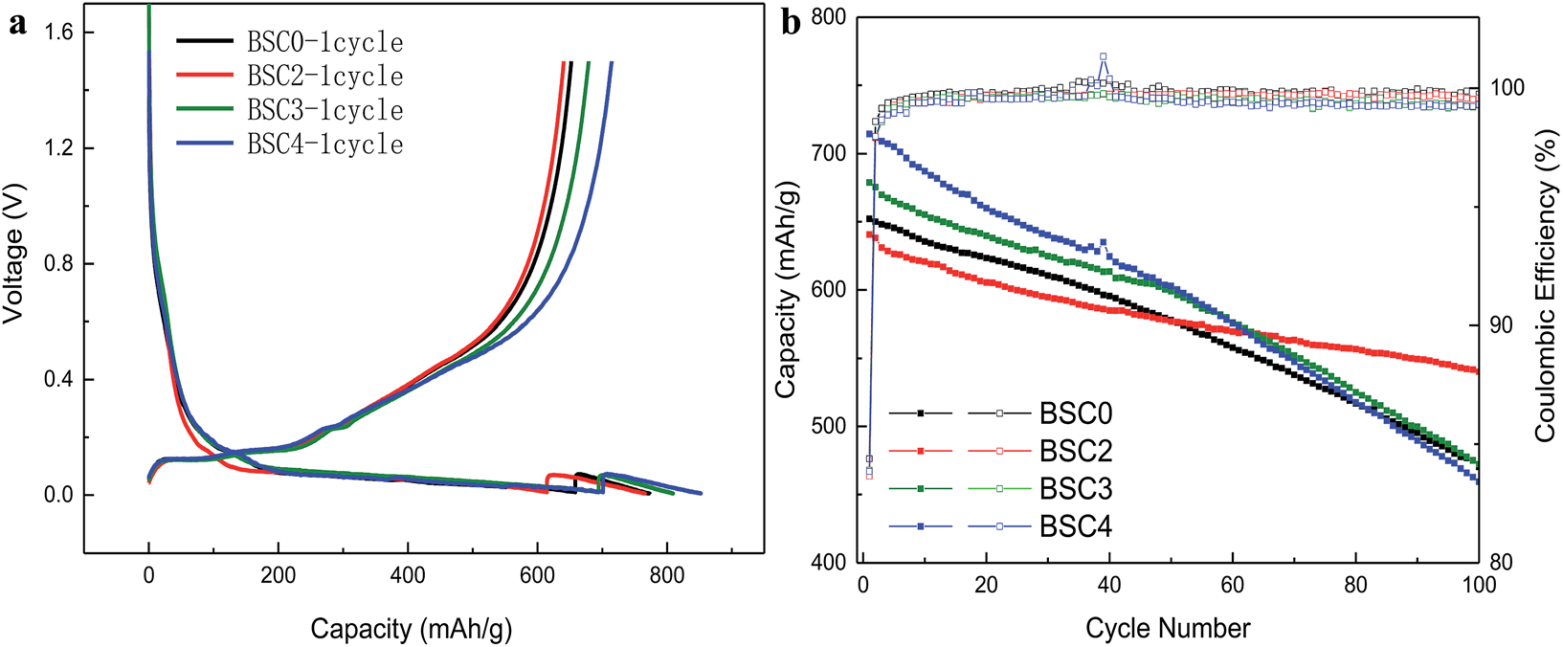
Electrochemical performances of SiO_*x*_/C materials with different PSDs in half cells at the charge/discharge rate of 0.1C/0.01C. Initial charge–discharge profiles (a) and cycling performances with CE plots for 100 cycles (b).

Fig. 4(a) demonstrates the initial charge–discharge profiles of the SiO_*x*_/C materials. The first lithium intercalation of BSC0 has discharge platforms of 0.15 V, 0.06 V and 0.03 V, and charge platforms of 0.13 V, 0.15 V, 0.23 V and 0.45 V. Among them, the long voltage platform near 0.15 V corresponds to the lithium and silicon alloying process^14^ which includes two processes: one is the formation of irreversible products Li_2_O and Li_4_SiO_4_, and the other is Li and silicon forming lithium silicon alloy process. The specific equations are:SiO_*x*_ + Li → Li_2_O + SiSiO_*x*_ + Li → Li_4_SiO_4_ + SiSi + Li → Li_*x*_Si

The oxidation peak near 0.45 V is related to the de-lithiation process of Li_15_Si_4_. The first lithium intercalation capacity and coulomb efficiency of BSC0 is 773 mA h g^−1^ and 84.38%, and the corresponding reversible specific capacity is 652.2 mA h g^−1^. The specific charge and discharge data are shown in Table 1. The first CEs of the four electrodes are ∼84% with no significant difference. Compared with BSC0, the first lithium intercalation capacity of BSC3 and BSC4 increase by 36.3 mA h g^−1^ and 79.1 mA h g^−1^, respectively, while the first lithium intercalation capacity of BSC2 is 7.1 mA h g^−1^ lower than that of BSC0. We could presume that with the reduction of particle size, the first lithium intercalation capacity of the corresponding electrode is increasing. The increase in the maximum specific intercalation capacity of the electrode may be due to the decrease in the number of large particles, resulting in shorter diffusion paths and larger specific surface area. This is consistent with the simulation results in ref. 15 which concluded that smaller average diameter of the material contributed to higher electrode specific capacity. We also notice that specific capacity of BSC0 is slightly above BSC2, indicating that for materials with almost equivalent particle size, wider particle size distribution contributes to higher electrode specific capacity. This is agreed with simulation results in ref. 16 which demonstrated that for materials with the same average particle radius, wider particle size distributions delivered higher energy density than monodisperse-sized particles for low C-rates. This phenomenon is probably owing to the faster lithiation of small particles which provides electrochemical potential gradients between particles. To further confirm the effect of the particle sizes of electrodes on the electrochemical properties, the cycling curves of SiO_*x*_/C electrodes with different material PSDs are shown in Fig. 4(b). The charge–discharge specific capacity and the coulomb efficiency are shown in Table 1, *Q*_discharge_ represents the discharge specific capacity, *Q*_charge_ represents the charge specific capacity, and the average coulomb efficiency is the average value of the cycling CEs from 2nd cycle to 100th cycle. The capacity retention of BSC0, BSC2, BSC3, BSC4 electrodes after 100 cycles are 470.2 mA h g^−1^, 540.1 mA h g^−1^, 472.1 mA h g^−1^ and 459.4 mA h g^−1^, respectively, corresponding to capacity retention ratio of 72.09%, 84.31%, 69.56% and 64.30%, respectively. The electrode capacity retention ratio shows a decreasing trend along with the decrease of micro-particle size. It is probably due to the increase of the specific surface area contributes to the growth of the contact area between silicon materials and electrolyte, resulting in more side reactions associated with the decomposition of electrolyte. And further leading to increased irreversible capacity, thus accelerating decrease of the reversible capacity. BSC0 electrode exhibits better performance than BSC2 in the first 40 cycles, but the capacity of BSC2 electrode tends to be more stable in the subsequent cycles, while the BSC0 electrode capacity still declines. The change in the electrode capacity retention rate indicates that the slope of the BSC2 curve is flatter while the deterioration degree of the BSC0 electrode is severer. After about 40 cycles, the decay rate of BSC0 capacity accelerates, leading to capacity retention only 72.09% after 100 cycles, which is significantly lower than that of BSC2 electrode (84.31%). This major difference between cycling stability is probably due to the fact of wider active materials PSD in BSC0 than that in BSC2 electrode. The underlying reason for this phenomenon is the presence of small particles contributing to longer diffusion path.

**Table 1:** Specific capacities and coulombic efficiencies of SiO_*x*_/C materials with different PSDs in half cells

Sample	Cycle no.	*Q*_discharge_ (mA h g^−1^)	*Q*_charge_ (mA h g^−1^)	Efficiency (%)	Cycle retention (%)	Average CE (%)
BSC0	1st	773	652.2	84.38	72.09	99.81
	100th	471.3	470.2	99.75		
BSC2	1st	765.9	640.6	83.65	84.31	99.63
	100th	542.4	540.1	99.58		
BSC3	1st	809.2	678.9	83.9	69.56	99.46
	100th	475.5	472.1	99.29		
BSC4	1st	852.1	714.5	83.85	64.3	99.43
	100th	462.6	459.4	99.31		

The cycling CE usually represents the actual consumption of lithium in the electrochemical cycles.^17–19^ Since the total amount of Li in the full cell is limited, the cycling CE in the half-cell has a certain significance to evaluate the overall battery capacity retention. As shown in Fig. 4(b) and Table 1, the cycling CE of BSC0 electrode grows the fastest, increasing above 99.5% only after 7 cycles, and maintaining this level in the following cycles. The average cycling CE from 2nd cycle to 100th cycle of the BSC0 electrode is 99.81%, which is significantly higher than those of the other groups. The cycling CE of BSC2 and BSC3 increase to 99.5% at 15th cycle and 12th cycle respectively, and the cycling CE of BSC4 electrode never reaches 99.5%. The average particle size of the active material used in the BSC4 electrode is the smallest and the lower CE may be caused by more sever side reactions. On the basis of these results, we could also conclude that the poor electrochemical performance of BSC0 with wider PSD does not result from the low cycling CE, but may be due to electrode structure failure caused by small particles.

**Fig. 5:**
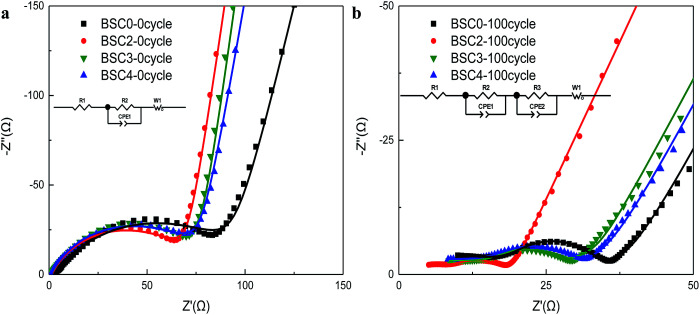
Nyquist plots of electrodes made of SiO_*x*_/C materials with different PSDs before cycling (a) and after 100 cycles (b) (dot: the experimental values; line: the fitted values).

To further investigate the possible effect of particle size distribution on the performance of cells, the EIS measurements on electrodes were executed before and after cycling, and the EIS results with the model are shown in Fig. 5. The impedance spectra of the electrodes before cycling are composed of a semicircle and a slash. The semicircle (at middle and high frequency regions) corresponds to the charge transfer impedance (*R*_ct_) at the electrode–electrolyte interface^20,21^ and the slash (at low frequency region) corresponds to the Warburg diffusion resistance (*Z*_w_) of Li^+^ in the solid electrode material.^22,23^ The flattened shape of semicircle may be caused by the porous structure and the surface roughness of the electrodes.^24^ Before cycling, although the slopes of BSC2, BSC3, BSC4 show no big difference, the radius of the semicircle increase according to the order of BSC2, BSC3, BSC4. The *R*_ct_ of BSC2, BSC3, BSC4 electrodes before cycling is 68.88 Ω, 75.86 Ω, and 76.24 Ω, respectively. The increase of radius of *R*_ct_ may be caused by the increase in the contact resistance of the material,^25^ that is, as the particle size decreases, the interfacial impedance of the electrode increases, which could be related to the specific surface area of the active materials. As the particle size decreases, the specific surface area increases and the corresponding interface impedance increases. In addition, the slope of the slash in the low frequency region of the BSC0 electrode is smaller than the slopes of other electrodes with narrow active material PSD. The small diffusion resistance of Li^+^ in the solid phase may be related to the enhancement electrical contact resulting from the close packing of the active material particles. The impedance spectra of the electrodes after cycling are composed of two semicircles and a slash, and the new semicircle at high frequency region corresponds to the SEI impedance (*R*_SEI_). The corresponding fitted impedance parameters are listed in Table S1.† The *R*_ct_ of BSC0, BSC2, BSC3, BSC4 electrodes after 100th cycles are 8.93 Ω, 6.52 Ω, 15.5 Ω, and 65.08 Ω, respectively, due to the modified equivalent circuit model given in the inset of Fig. 5(b). The decrease of *R*_ct_ after cycling may be owing to the activation of electrode materials and the full infiltration of electrolyte into electrode materials.^26^ Apparently, the BSC2 electrode has the lowest *R*_ct_, which can be ascribed to the well maintained SiO_*x*_/C electrode structure, thus the best cycling performance was achieved. The *R*_ct_ of BSC0 electrode is much higher than that of BSC2 electrode, indicating the worse conductivity which may be probably caused by electrode structure failure in the late cycling period.

### 3.3. Electrode structural testing of fabricated electrodes

**Fig. 6:**
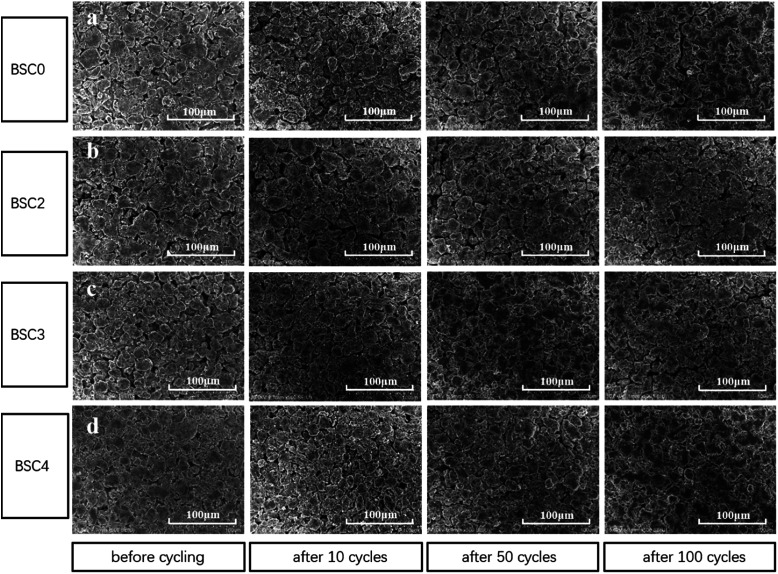
Surface SEM images of (a) BSC0, (b) BSC2, (c) BSC3, (d) BSC4 electrode before cycling and after 10, 50, 100 cycles.

The porosity of BSC0, BSC2, BSC3 and BSC4 electrodes before cycling was measured by mercury intrusion method and the results are 48.54%, 56.54%, 47.26% and 42.65% respectively. It is clear that with the decrease of the active material size, the porosity reduces. Due to the filling effect in the BSC0 electrode, the porosity of BSC0 electrode is lower than that of BSC2. To further study the structural properties of the electrodes with different active particle dimensions, the electrodes before and after the cycling were observed by SEM (as shown in Fig. 6). Fig. 6(a–d) correspond to BSC0, BSC2, BSC3, BSC4 samples respectively. The spherical particles in the pristine electrodes show smooth surface, no significant cracks. The minor cracks on the surface of individual particles may result from the rolling process. The active material particle size in the BSC4 sample is the smallest, thus the contact between the particles is most tense, and the pore size is only ∼3 μm. Comparing Fig. 6(a) and (b), the BSC0 electrode structure is more compact than BSC2, it is because the PSD of the BSC0 material is wide and the small particles are filled in the pores of the other particles (filling effect) to reduce the number and size of pores. The spherical structures of several groups of SiO_*x*_/C still exist after 10 cycles compared with that before cycling. The roughness of the material surface is improved probably because the active materials react with electrolyte at the solid–liquid interface to form SEI. The whole electrode surfaces show no obvious cracks, indicating that due to the existence of buffer space, material structure and electrode structure integrity could be maintained during early cycles to a certain extent. As the number of cycles increase, the cracks on the electrode surface grow gradually. BSC0, BSC3 and BSC4 show similar surface morphology changes. At 50th cycle, there are no obvious cracks on the electrode surface, while cracks of ∼5 μm width appear after 100 cycles. The BSC2 electrode with most stable cycling performance shows almost the same surface before and after cycling 100 cycles, there is no obvious cracks and the pores between the active material particles shrink slightly, indicating that the pores existing in the BSC2 electrode are effective during long term cycle. The proper pore structure is crucial to maintain the integrity of the electrode structure as well as the material structure, and exhibit excellent electrochemical performance. Statistical analysis of the particle size distribution of BSC0–BSC4 electrode samples before cycling is obtained by Nano Measurer analysis software according to the electrode surface SEM images (Fig. 6), and the statistical results are 20.91 μm, 22.08 μm, 17.77 μm, 13.96 μm, respectively. After 100th lithiation, the statistical values are 20.84 μm, 22.47 μm, 17.47 μm, 14.01 μm, respectively, almost the same as those before cycling. We could conclude that due to the generation of SEI, the morphology of active materials becomes rougher and the particle boundary becomes more blur, while the size of the active material particles does not change significantly after long term cycling. We could speculate that the difference in electrode cycling performance may result from: (1) the growth of the SEI and (2) whether the void space between particles could accommodate the volume change of active materials effectively, preventing the electrode from cracking and rupturing.

**Fig. 7:**
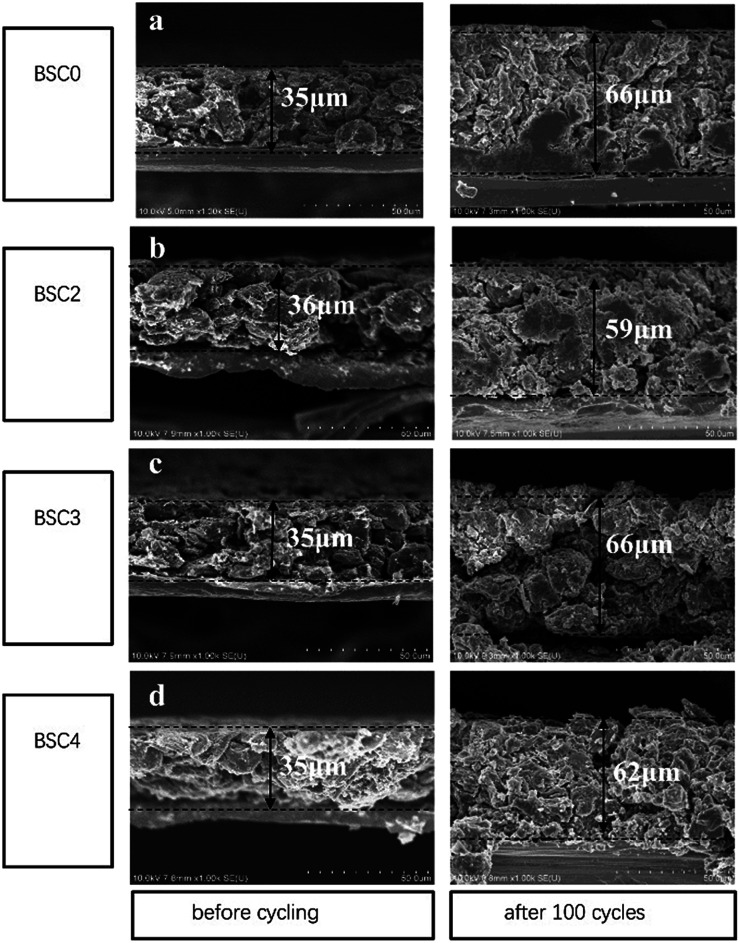
Cross sectional SEM images of (a) BSC0, (b) BSC2, (c) BSC3, (d) BSC4 electrode before cycling and after 100th lithiation.

The cross-sectional SEM images of electrodes with different active particle sizes are shown in Fig. 7. The current collector copper foil has a thickness of ∼8 μm. After 100 cycles, the electrode sheets expand to a certain degree in the direction perpendicular to the current collector, but the current collector still maintains good electrical contact with the active materials. The thickness of BSC0, BSC2, BSC3 and BSC4 electrodes before the cycling is 35 μm, 36 μm, 35 μm and 35 μm respectively. After 100 cycles, the thickness of the electrode sheets increases to 66 μm, 59 μm, 66 μm and 62 μm respectively, with the percentages of increment being 65%, 58%, 65% and 77%, respectively. Apparently, with the decrease of the particle size, the volume expansion rate of the corresponding electrode tends to increase over 100 cycles, which may be related to the more intense intercalation and deintercalation reaction caused by larger specific surface area of the active materials. BSC2 electrode displays the smallest volume expansion in the three groups of narrow particle size distribution, which may be due to the smaller specific surface area and larger pores of accumulation between particles, thus providing effectively buffering ability. This phenomenon is consistent with the BSC3 and BSC4 electrodes suffer capacity decreasing in varying degrees during the late cycles, showing the gradually increase of electrode polarization, while BSC2 electrode has better cycle stability. The gradually shrink and block of the electrode pores during the late cycles will cause the reduction of the void space and then results in insufficient space to buffer the volume change of the active materials. Finally leading to the destruction of the material structure or the electrode structure. With the reducing of active material particle size, the available space for electrode is more limited. In addition, BSC0 electrode shows a larger change in thickness of the electrode compared to BSC2 electrode, with a volumetric expansion rate of 65% over 100th cycles, indicating that the space utilization of the BSC0 electrode with wider particle size distribution is also less sufficient. After 100th cycles, the pore size in the BSC0 electrode significantly reduces, mainly in the range of 1–2 μm (Fig. 7(a)). While there is still effective buffer space of ∼5 μm in the BSC2 electrode (Fig. 7(b)). Combined with the change of electrode morphology and electrochemical performance, it could be speculated that the main reason for BSC0 electrode is inferior to BSC2 electrode in cycle stability may be cracks generated in the late stage of cycling. The insufficient buffer space affects the electrical contact between active materials, leading to faster specific capacity decay than BSC2 electrode.

## 4. Conclusion

In summary, we obtained micro-sized SiO_*x*_/C with different PSDs by mechanical sieving, and electrochemical tests show that the initial reversible specific capacity of the electrodes tends to increase as the particle size decreases, while cycle stability becomes poor. The decrease of large particles and the increase of specific surface area corresponds to larger reactive sites as well as larger irreversible capacity caused by side reactions. For electrodes made of similar *D*_50_ but different PSD width, the broadening of the particle size distribution may contribute to higher energy density and better volumetric capacity, but at the same time leads to more severe degree of electrode polarization. For wider PSD electrode, it is probably the electrode degradation caused by the limited buffer space but not the low cycling CE that results in the inferior electrochemical performance compared to electrodes with narrower PSD. For the micro-sized SiO_*x*_/C material, when testing at the areal density of 4 mg cm^−2^, the optimized particle size *D*_50_ and *D*_90_/*D*_50_ are 22.7 μm and 1.59, respectively. The corresponding optimized electrode shows stable cycling performance of 100 cycles at 0.1C, with the capacity retention of 84.31% and the average coulombic efficiency of 99.63%.

## Conflicts of interest

There are no conflicts to declare.

## Supplementary Material

RA-008-C8RA00539G-s001
