# Correction: Effect of particle size distribution on the electrochemical performance of micro-sized silicon-based negative materials

**DOI:** 10.1039/c8ra90045k

**Published:** 2018-05-30

**Authors:** Shuaijin Wu, Bing Yu, Zhaohui Wu, Sheng Fang, Bimeng Shi, Juanyu Yang

**Affiliations:** General Research Institute for Nonferrous Metals Beijing 100088 China juanyuyang@163.com; China Automotive Battery Research Institute Co., Ltd. Beijing 100088 China

## Abstract

Correction for ‘Effect of particle size distribution on the electrochemical performance of micro-sized silicon-based negative materials’ by Shuaijin Wu *et al.*, *RSC Adv.*, 2018, **8**, 8544–8551.

There was a small error in the funder number of the first funder acknowledged and the correct version of the acknowledgements is shown below.

The work was supported by the National Key R & D Program of China (No. 2016YFB0100400) and the National Natural Science Foundation of China (No. 51504032, 51604032, U1664256).

The Royal Society of Chemistry apologises for these errors and any consequent inconvenience to authors and readers.

## Supplementary Material

