# Supplementary material for: Effect of particle size distribution on the electrochemical performance of micro-sized silicon-based negative materials
Source: RSC Adv. 2018 Feb 23;8(16):8544–51. doi: 10.1039/c8ra00539g (PMC9078586; doi:10.1039/c8ra00539g)
Supplement: RA-008-C8RA00539G-s001 [file RA-008-C8RA00539G-s001.pdf]

Electronic Supplementary Material (ESI) for RSC Advances.
This journal is © The Royal Society of Chemistry 2018

Supplementary Information

Effect of Particle Size Distribution on the Electrochemical Performance of Micro-sized Silicon-based Negative Materials

Shuaijin Wu, [ab] Bing Yu, [b] Zhaohui Wu, [b] Sheng Fang,[b] Bimeng Shi, [b] and Juanyu Yang * [ab]

[a.] General Research Institute for Nonferrous Metals, Beijing 100088, China

[b.] China Automotive Battery Research Institute Co., Ltd, Beijing 100088, China

Table S1 Impedance parameters for SiOx/C electrodes with different PSDs after 100 cycles.

Electrode	R_{SEI}/Ω	CPE1-T/F	CPE1-P	R_{ct}/Ω	CPE2-T/F	CPE2-P	Z_w-R/Ω
BSC0	18.53	2.00E-05	0.80112	8.933	6.27E-04	0.78464	5.3335
BSC2	8.902	8.37E-05	0.58055	6.519	1.41E-04	0.59933	6.009
BSC3	17.634	4.40E-05	0.39208	15.5	4.43E-04	0.78657	19.85
BSC4	26.623	2.65E-05	0.41246	65.08	3.96E-04	0.82096	7.6899
